# Effect of Triazole Fungicides Titul Duo and Vintage on the Development of Pea (*Pisum sativum* L.) Symbiotic Nodules

**DOI:** 10.3390/ijms24108646

**Published:** 2023-05-12

**Authors:** Artemii P. Gorshkov, Pyotr G. Kusakin, Yaroslav G. Borisov, Anna V. Tsyganova, Viktor E. Tsyganov

**Affiliations:** 1 Laboratory of Molecular and Cell Biology, All-Russia Research Institute for Agricultural Microbiology, Saint Petersburg 196608, Russia; a.gorshkov@arriam.ru (A.P.G.); pyotr.kusakin@arriam.ru (P.G.K.); avtsyganova@arriam.ru (A.V.T.); 2 Research Resource Centre “Molecular and Cell Technologies”, Saint Petersburg State University, Saint Petersburg 199034, Russia; gis.ib88@gmail.com; 3 Saint Petersburg Scientific Center RAS, Universitetskaya Embankment 5, Saint Petersburg 199034, Russia

**Keywords:** *Pisum sativum* L., symbiotic nodule, symbiosome, bacteroid, cell wall, fungicide

## Abstract

Triazole fungicides are widely used in agricultural production for plant protection, including pea (*Pisum sativum* L.). The use of fungicides can negatively affect the legume-*Rhizobium* symbiosis. In this study, the effects of triazole fungicides Vintage and Titul Duo on nodule formation and, in particular, on nodule morphology, were studied. Both fungicides at the highest concentration decreased the nodule number and dry weight of the roots 20 days after inoculation. Transmission electron microscopy revealed the following ultrastructural changes in nodules: modifications in the cell walls (their clearing and thinning), thickening of the infection thread walls with the formation of outgrowths, accumulation of poly-β-hydroxybutyrates in bacteroids, expansion of the peribacteroid space, and fusion of symbiosomes. Fungicides Vintage and Titul Duo negatively affect the composition of cell walls, leading to a decrease in the activity of synthesis of cellulose microfibrils and an increase in the number of matrix polysaccharides of cell walls. The results obtained coincide well with the data of transcriptomic analysis, which revealed an increase in the expression levels of genes that control cell wall modification and defense reactions. The data obtained indicate the need for further research on the effects of pesticides on the legume-*Rhizobium* symbiosis in order to optimize their use.

## 1. Introduction

The biotic stresses (e.g., pests, diseases, and weeds) are important factors limiting plant growth and agricultural production. Among them, fungal diseases have reduced the world’s crop yields by almost 20% [1]. Root rot, seedling rot, rust, and powdery mildew lead to partial or complete crop loss [2,3,4,5,6]. The crops in Russia are most susceptible to Fusarium root rot, the losses are up to 50%, and in some years, the crop may die completely as a result of the disease [7]. The application of chemical fungicides is considered the main method of protecting crops from many diseases due to their convenience and low costs [8].

Combined triazole fungicides Titul Duo (propiconazole 200 g/L + tebuconazole 200 g/L) and Vintage (difenoconazole 65 g/L + flutriafol 25 g/L) are used in complex protection of legume crops in Russia [9]. When spraying, the preparations are sorbed by leaves and stems, penetrating the plant tissues. Triazoles, upon penetration into phytopathogenic fungi, inhibit the enzyme lanosterol 14α-demethylase, which is necessary for the biosynthesis of ergosterol, the main sterol in many fungal species [10,11], which leads to their impaired growth and death [12,13].

Different triazole compounds, such as triadimefon, propiconazole, hexaconazole, and paclobutrazol, are widely used as fungicides, and they can influence plant growth [14,15,16,17]. Triazoles act as plant growth regulators and affect hormonal balance, photosynthesis rate, enzyme activity, lipid peroxidation, and yield in various crops [14,18,19,20]. In particular, triazoles inhibit cytochrome P450-mediated oxidative demethylation, as well as the conversion of kaurene to kaurenoic acid, in the gibberellin biosynthetic pathway [14]. Triazoles cause morphological (stimulation of root growth and inhibition of shoot elongation) and biochemical (increased cytokinin synthesis and a temporary increase in abscisic acid) changes [14,18]. In addition, due to their inherent induction of an efficient free radical scavenging system that allows plants to detoxify reactive oxygen species (ROS), triazole compounds are sometimes used as stress protectants [21,22,23,24,25,26].

Pea (*Pisum sativum* L.) is one of the main legume crops in the world [27]. Like other legumes, pea forms a symbiotic relationship with *Rhizobium leguminosarum* bv. *viciae*. Rhizobia can contribute to overcoming the negative effects of pesticides. They secrete siderophores [28,29], produce 1-aminocyclopropane-1-carboxylate (ACC) deaminase that catalyzes ACC (precursor of ethylene) degradation [30,31], and solubilize insoluble phosphorus [32]. Therefore, the effects of fungicide treatment should be considered in terms of legume-*Rhizobium* symbiosis [33,34].

Despite the visible positive effects of triazoles on various crops [35,36,37,38,39,40], phytotoxic effects were reported [41,42,43,44,45]. Particularly, strong effects were manifested during the development of legume-*Rhizobium* symbiosis [41,42,46]. The use of tebuconazole caused a decrease in the number and weight of nodules, as well as the dry weight of the roots and shoots of pea plants [42]. In another study, tebuconazole significantly reduced the biomass of roots and shoots in pea, lentil (*Lens esculenta* Moench), mungbean (*Vigna radiate* L. (R) Wilczek), and chickpea (*Cicer arietinum* L.) plants by an average of 30% compared with controls, and also reduced the nodule number, with a maximum decrease of 67% in pea [41].

In this study, the effects of two triazole fungicides (Titul Duo and Vintage) on morphological and transcriptomic changes in pea symbiotic nodules were investigated. As a result, the dose-dependent negative effects of Titul Duo and Vintage treatment on legume-*Rhizobium* symbiosis were revealed. The influence of the stage of plant development when fungicides were applied was also shown.

## 2. Results

### 2.1. Nodulation and Plant Growth Parameters

**Figure 1:**
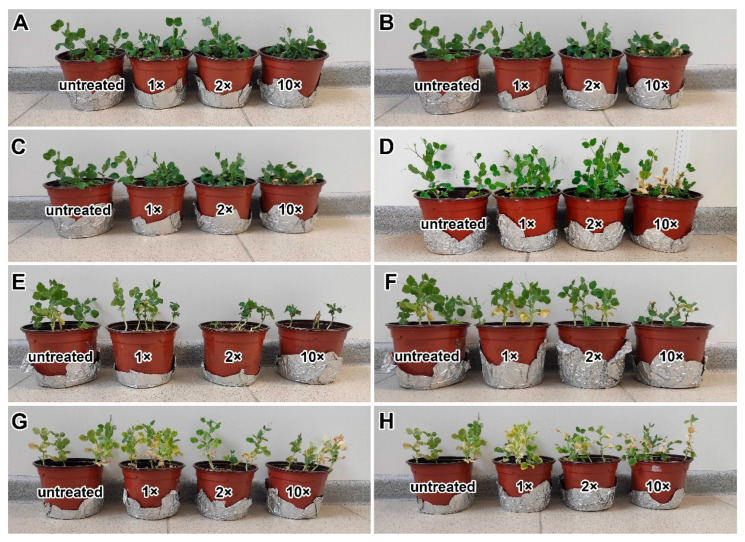
Phenotypes of pea plants (*Pisum sativum* L.) of the cultivars ‘Finale’ (**A**–**D**) and ‘Frisson’ (**E**–**H**). Untreated plants and plants treated with recommended by the manufacturer (1×), double-(2×), and tenfold-concentrated (10×) solutions of Titul Duo (**A**,**C**,**E**,**G**) and Vintage (**B**,**D**,**F**,**H**). (**A**,**B**,**E**,**F**) Plants treated at 10 DAI. (**C**,**D**,**G**,**H**) Plants treated at 20 DAI.

Both fungicides affected the plant growth of the pea cultivars ‘Finale’ and ‘Frisson’. In the 20- and 30-day-old plants, the shoot height of the treated plants decreased, they became thinner, and the leaves turned yellow in a dose-dependent manner (Figure 1A–H). Treatment at 10 days after inoculation (DAI) with both the double- and tenfold-concentrated solutions of the fungicide Titul Duo caused the strong inhibition of plant growth in the cv. ‘Frisson’ (Figure 1E). 

**Figure 2:**
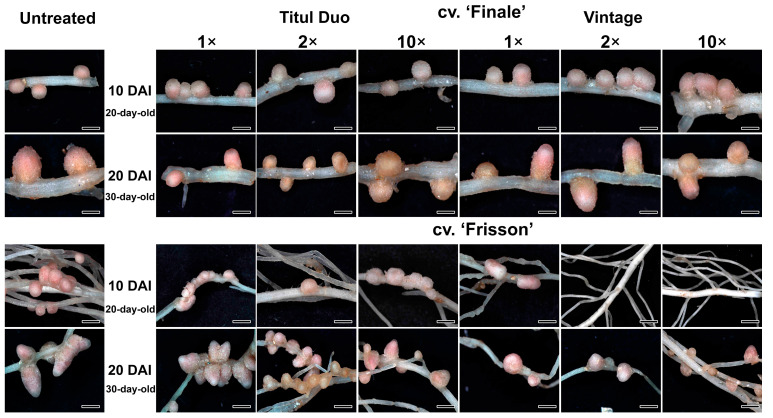
Nodule phenotypes of pea plants (*Pisum sativum* L.) of the cultivars ‘Finale’ and ‘Frisson’. Untreated plants and plants treated with recommended by the manufacturer (1×), double-(2×), and tenfold-concentrated (10×) solutions of Titul Duo and Vintage. Bars = 1 mm.

Nodules of plants treated with both fungicides did not differ from untreated ones by color (Figure 2). However, plants of cv. ‘Frisson’ formed a decreased number of nodules or did not form nodules at all when treated at 10 DAI with the double- and tenfold-concentrated solutions of fungicide Vintage (Figure 2). 

**Figure 3:**
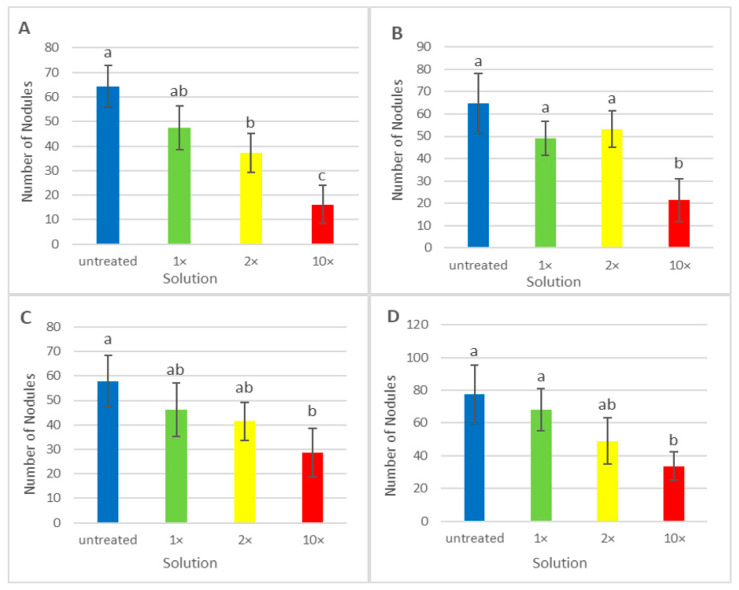
Mean nodule number per plant of pea (*Pisum sativum* L.) cv. ‘Frisson’ treated with recommended by the manufacturer (1×), double-(2×), and tenfold-concentrated (10×) solutions of fungicides Titul Duo (**A**,**C**) and Vintage (**B**,**D**). (**A**,**B**) Fungicide treatment at 10 DAI. (**C**,**D**) Fungicide treatment at 20 DAI. Different letters indicate groups with a significant difference according to the least significant difference test (*p* < 0.05; *n* = 20). Vertical bars represent standard deviation.

Growth and nodule parameters were measured for plants of the cv. ‘Frisson’ treated with both fungicides. Nodule numbers followed this trend: untreated >1× > 2× > 10×, except for Vintage at 10 DAI, where the trend was untreated >2× > 1× > 10×. However, only the difference between the untreated and the 10× was statistically different (Figure 3). When treated with various concentrations of fungicides, no statistically significant differences were found in the dry mass of shoots at both treatment time points (Supplementary Materials Figure S1A–D). Only treatment with fungicides at the highest concentration at 10 DAI reduced the dry weight of the roots (Supplementary Materials Figure S2A–D).

### 2.2. Nodule Histological Organization

**Figure 4:**
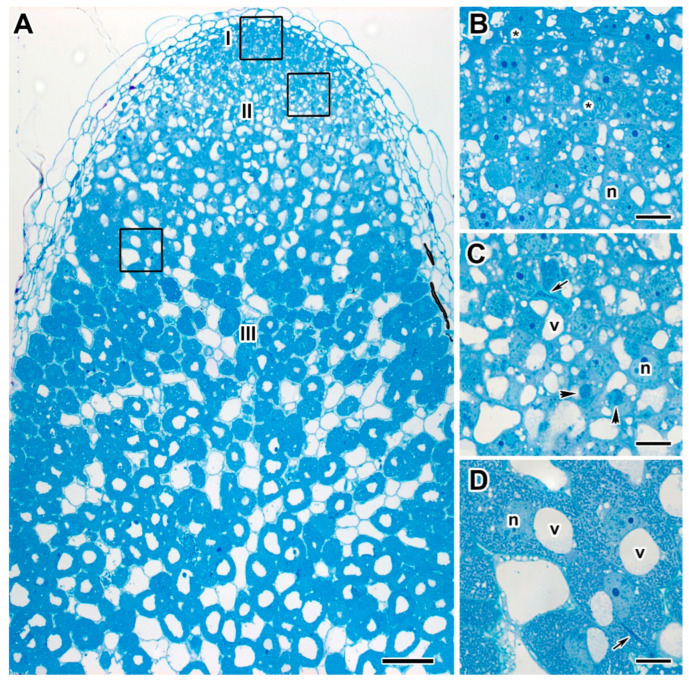
Histological organization of nodules of 20-day-old untreated plants of pea (*Pisum sativum* L.) cv. ‘Frisson’. (**A**) Longitudinal section of a nodule. (**B**–**D**) High magnification of the boxed area in (**A**). (**B**) Nodule meristematic cells. (**C**) Cells in the infection zone. (**D**) Infected cells in the nitrogen fixation zone. I, meristem; II, infection zone; III, nitrogen fixation zone; n, nucleus; v, vacuole; *, metaphase plate. Arrows indicate infection threads; arrowheads indicate infection droplets. Bars (**A**) = 100 µm, (**B**–**D**) = 10 µm.

The detailed analysis of both 20- and 30-day-old nodules of untreated plants of the cv. ‘Frisson’ (Figure 4A) showed a histological organization typical for an indeterminate nodule. Meristematic cells had numerous small vacuoles, a large nucleus with a nucleolus, and an electron-dense cytoplasm (Figure 4B). Metaphase plates were often seen (Figure 4B). Numerous infection threads and droplets were present in the infection zone, and a few juvenile bacteroids were located along the cell periphery (Figure 4C). Mature nitrogen-fixing cells with a central vacuole were filled with numerous pleomorphic bacteroids (Figure 4D).

**Figure 5:**
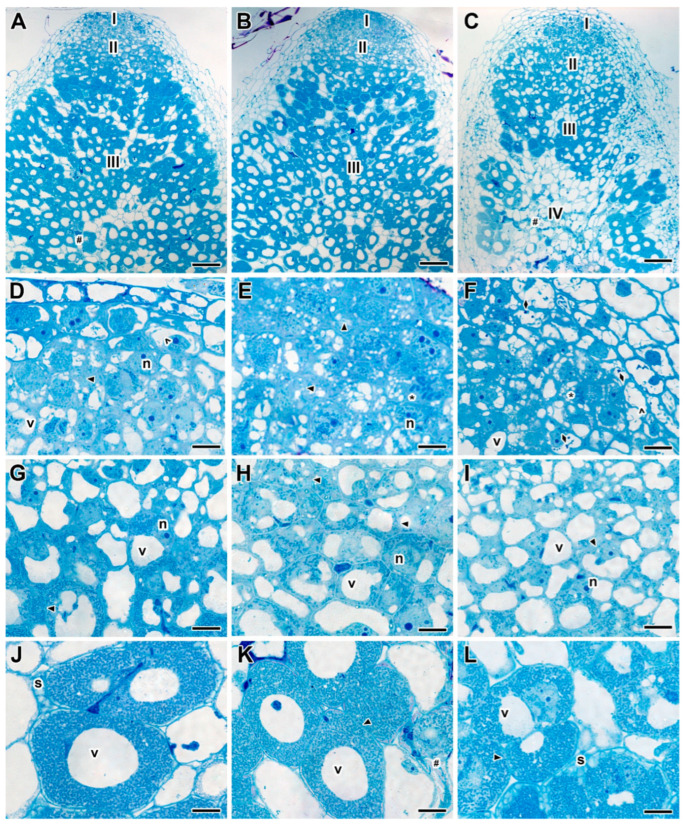
Histological organization of the nodules of pea (*Pisum sativum* L.) cv. ‘Frisson’ treated with fungicide Titul Duo at 10 DAI. (**A**,**D**,**G**,**J**) Treatment with fungicide at the concentration recommended by the manufacturer. (**B**,**E**,**H**,**K**) Treatment with a double-concentrated solution of fungicide. (**C**,**F**,**I**,**L**) Treatment with a tenfold-concentrated solution of fungicide. (**A**–**C**) Longitudinal section of a nodule. (**D**–**F**) Nodule meristematic cells. (**G**–**I**) Cells in the infection zone. (**J**–**L**) Infected cells in the nitrogen fixation zone. I, meristem; II, infection zone; III, nitrogen fixation zone; IV, senescence zone; n, nucleus; v, vacuole; *, metaphase plate; #, degrading cell; s, abnormal accumulation of starch in infected cells in the nitrogen fixation zone. Triangles indicate a barely visible cell wall between infected cells; rhombi indicate inclusions in vacuoles; empty arrowheads indicate vacuole fusion. Bars (**A**–**C**) = 100 µm, (**D**–**L**) = 10 µm.

Plants of cv. ‘Frisson’ treated with the different concentrations of fungicide Titul Duo at 10 DAI demonstrated clearly visible abnormalities in the histological zones of nodules (Figure 5A–C). Meristematic cells had a folded cell surface; small vacuoles merged into large vacuoles (Figure 5D–F). At the highest concentration of fungicide in the vacuoles of meristematic cells, dark inclusions, presumably, phenolic compounds, were observed (Figure 5F). The cell walls in the meristem and the infection zone were curved and sometimes thinned; in such places, it was difficult to distinguish cell boundaries (Figure 5D–I). These effects of Titul Duo intensified with increasing concentrations. In the nitrogen fixation zone, the boundaries of infected cells sometimes were barely visible (Figure 5K,L); however, such cells were less common when compared with the meristem and the infection zone. In addition, numerous degenerating cells were seen (Figure 5K). At the highest concentration of fungicide, cells in the nitrogen fixation zone contained an increased amount of starch granules (Figure 5L). Even more, a senescence zone was formed at the base of the nodule, which occupied more than half of the nodule (Figure 5C).

Treatment with the fungicide Vintage at 10 DAI caused similar abnormalities to those induced with Titul Duo treatment (Supplementary Materials Figure S3). At the highest fungicide concentration, there were nodules where the senescence zone occupied the entire tissue of the nodule (Supplementary Materials Figure S3C).

There were no pronounced differences in the types of abnormalities caused by the treatment with fungicides Titul Duo and Vintage at 10 and 20 DAI. However, the histological structure of nodules of the 30-day-old plants treated with both fungicides was less damaged (Supplementary Materials Figure S4A–C). Meristematic cells also had folded edges and small vacuoles fused into large vacuoles (Supplementary Materials Figure S4D–F). Degenerating cells were seen in the meristem (Supplementary Materials Figure S4D–F), as well as in the nitrogen fixation zone (Supplementary Materials Figure S4J–L). The cells of the meristem and the infection zone had a folded cell surface, but to a lesser extent in comparison with the treatment with fungicides at 10 DAI (Supplementary Materials Figure S4D–I). In some cells in the nitrogen fixation zone, the tonoplast was destroyed (Supplementary Materials Figure S4J–L). The cell walls in all zones in individual cells were thinned; the cell boundaries were barely distinguishable (Supplementary Materials Figure S4). The dose-dependent differences in abnormalities caused by fungicide treatment at 20 DAI were not revealed.

### 2.3. Ultrastructure of Nodules

**Figure 6:**
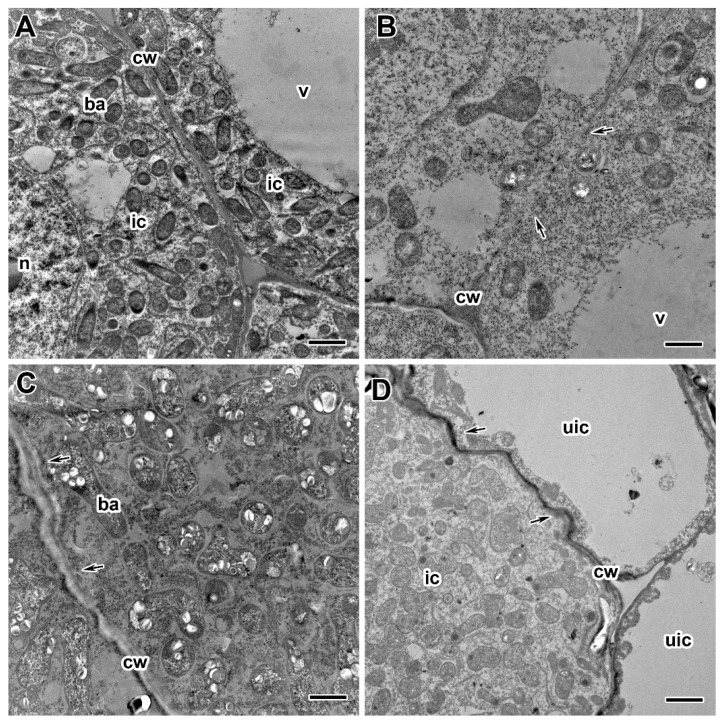
Ultrastructural organization of cell walls in nodules of pea (*Pisum sativum* L.) cultivars ‘Finale’ (**A**–**C**) and ‘Frisson’ (**D**). (**A**) Section of a nodule of an untreated 30-day-old plant. Treatment with fungicides Titul Duo (**B**,**D**) and Vintage (**C**) at 20 DAI at a concentration recommended by the manufacturer (**B**,**D**) and with a double-concentrated solution of fungicide (**C**). n, nucleus; v, vacuole; ic, infected cell; cw, cell wall; ba, bacteroid; uic, uninfected cell. Arrows indicate cell wall abnormalities. Bars (**D**) = 5 µm, (**A**,**C**) = 2 µm, (**B**) = 1 µm.

A comparative analysis of the ultrastructure of nodules of the two pea genotypes was carried out. Nodules of 20- and 30-day-old plants of cultivars ‘Finale’ and ‘Frisson’ grown without fungicide treatment had a similar ultrastructural organization characteristic of indeterminate nodules [47,48]. In the nitrogen fixation zone, in infected cells, numerous symbiosomes containing a single pleomorphic bacteroid were observed (Figure 6A). The cell walls in the entire tissue of the nodule were smooth and had a pronounced middle lamella (Figure 6A).

Treatment with fungicides Titul Duo and Vintage led to significant changes in the ultrastructure of nodules of both pea genotypes, and the severity of the changes depended on the concentration of fungicides and the time of treatment. The genotype-specific differences in abnormalities in nodule ultrastructure caused by the fungicide treatment were not revealed. 

The most striking abnormalities in the nodule ultrastructure after treatment of plants with fungicides were changes in the cell wall structure (Figure 6B–D). The cell walls in the meristem and the infection zone were most susceptible to the negative effect of fungicides. When treated with fungicides at the recommended concentration, the cell walls were curved and could also be less electron-dense (Figure 6C) or vice versa electron-dense (Figure 6D). These negative effects were more pronounced at the highest fungicide concentrations. However, in some cells, the cell wall became thinner, resulting in the cell borders being barely visible (Figure 6B).

**Figure 7:**
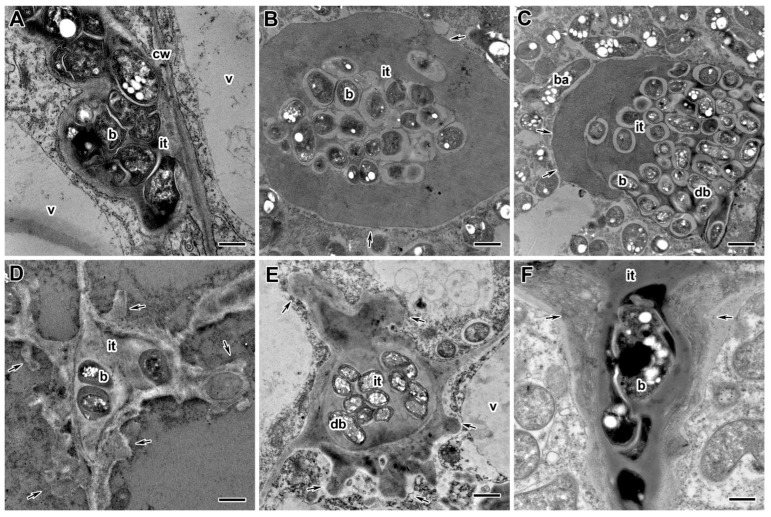
Ultrastructural organization of infection threads in nodules of the pea (*Pisum sativum* L.) cultivars ‘Frisson’ (**A**,**D**–**F**) and ‘Finale’ (**B**,**C**). (**A**) Section of a nodule of an untreated 30-day-old plant. Fungicide treatment with Titul Duo (**B**–**D**) and Vintage (**E**,**F**) at 10 DAI (**B**–**E**) and 20 DAI (**F**) at concentrations recommended by the manufacturer (**D**,**F**), and a tenfold-concentrated solution (**B**,**C**,**E**). it, infection thread; v, vacuole; cw, cell wall; ba, bacteroid; b, bacterium; db, degenerative bacterium. Arrows indicate infection thread wall abnormalities. Bars (**A**,**C**) = 2 µm, (**B,D**–**F**) = 1 µm.

Electron microscopy analysis revealed significant differences in the infection thread structure of nodules between untreated (Figure 7A) and fungicide-treated plants (Figure 7B–F). In nodules of fungicide-treated plants, the infection thread wall became less electron-dense (Figure 7B,C), and its outgrowths propagated in the cytoplasm (Figure 7D,E). In some infection threads, the wall was thickened and swelled (Figure 7F). Fungicide treatment at 20 DAI led to the formation of numerous fibrous layers in the infection thread wall (Figure 7F). Only at the highest concentrations of bacteria inside the infection threads underwent degenerative changes (Figure 7C,E). 

**Figure 8:**
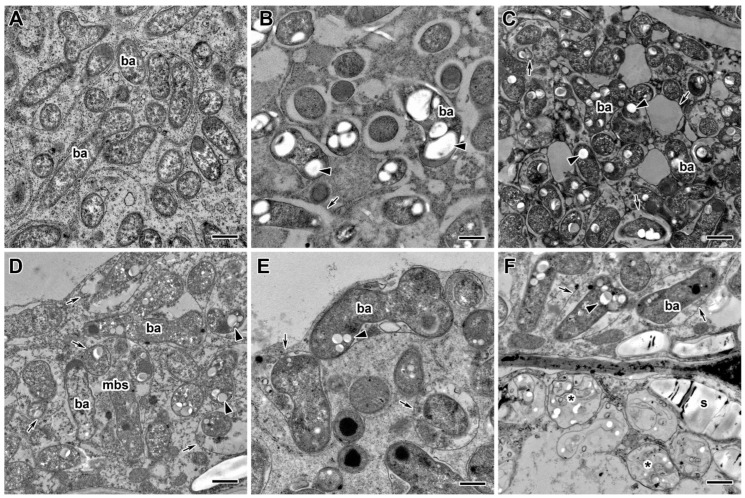
Ultrastructural organization of bacteroids in infected cells in nodules of the pea (*Pisum sativum* L.) cultivars ‘Frisson’ (**A**,**D**–**F**) and ‘Finale’ (**B**,**C**). (**A**) Untreated 30-day-old plants. Fungicide treatment of Titul Duo (**E**,**F**) and Vintage (**B**–**D**) at 10 DAI (**B**) and 20 DAI (**C**–**F**) at concentrations recommended by the manufacturer (**C**,**F**), with a double-concentrated solution (**E**) and a tenfold-concentrated solution (**B**,**D**). ba, bacteroid; *, “ghost” bacteroid; mbs, multibacteroid symbiosome; s, starch. Arrows indicate changes in the symbiosome membrane; arrowheads indicate poly-β-hydroxybutyrate drops in bacteroids. Bars (**C**) = 2 µm, (**A**,**B**,**D**–**F**) = 1 µm.

Ultrastructural analysis revealed various morphological changes in bacteroids in the infected cells in nodules of fungicide-treated pea plants (Figure 8B–F) in comparison to untreated plants (Figure 8A). At the concentration recommended by the manufacturer, the accumulation of poly-β-hydroxybutyrates (PHB) was observed in bacteroids (Figure 8C,F). In some cells at these concentrations, the expansion of the peribacteroid space was visible (Figure 8C–F). In some cells, symbiosomes, as a result of membrane fusion, contained several bacteroids at different stages of degeneration (Figure 8C,D). In addition, the symbiosome transformation into lytic compartments appeared (Figure 8D,E). Treatment with fungicides caused the degeneration of infected cells filled with “ghosts” of bacteroids in the nitrogen fixation zone of nodules (Figure 8F).

**Figure 9:**
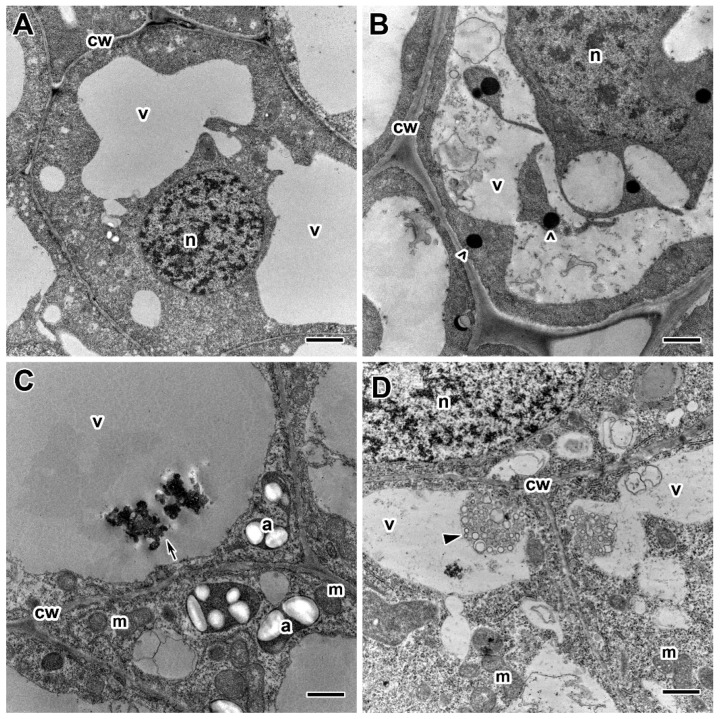
Ultrastructural organization of vacuoles in the nodules of the pea (*Pisum sativum* L.) cultivars ‘Frisson’ (**A**,**D**) and ‘Finale’ (**B,C**). (**A**) Section of a nodule of an untreated 20-day-old plant. Treatment with recommended by the manufacturer concentrations of fungicides Titul Duo (**D**) and Vintage (**B**,**C**) at 20 DAI. n, nucleus; v, vacuole; cw, cell wall; a, amyloplast; m, mitochondrion. Arrows indicate inclusions in vacuoles, arrowheads indicate multivesicular bodies, empty arrowheads indicate inclusions in meristematic cells, presumably of a phenolic nature. Bars = 2 µm.

Fungicide treatment of pea plants led to the formation of abnormalities associated with the vacuole (Figure 9B–D) in comparison to untreated plants (Figure 9A). In meristematic cells, numerous small vacuoles merge into one (Figure 9B). In some cells, the tonoplast formed numerous invaginations and vesicles into the central vacuole, which led to the appearance of multivesicular bodies of various sizes (Figure 9D). Some vacuoles contained inclusions of unclear composition (Figure 9C). At high concentrations of fungicides, multivesicular bodies were observed in almost every cell in the infection and nitrogen fixation zones.

In spite of observed abnormalities, fungicides did not affect the ultrastructure of plastids and mitochondria (Figure 9C,D). In addition, the use of fungicides resulted in earlier and more abundant starch accumulation, which is an indicator of an ineffective symbiosis (Figure 8F). Meristematic cells accumulated inclusions of presumably phenolic nature (Figure 9B). 

### 2.4. Immunocytochemical and Histochemical Analyses

**Figure 10:**
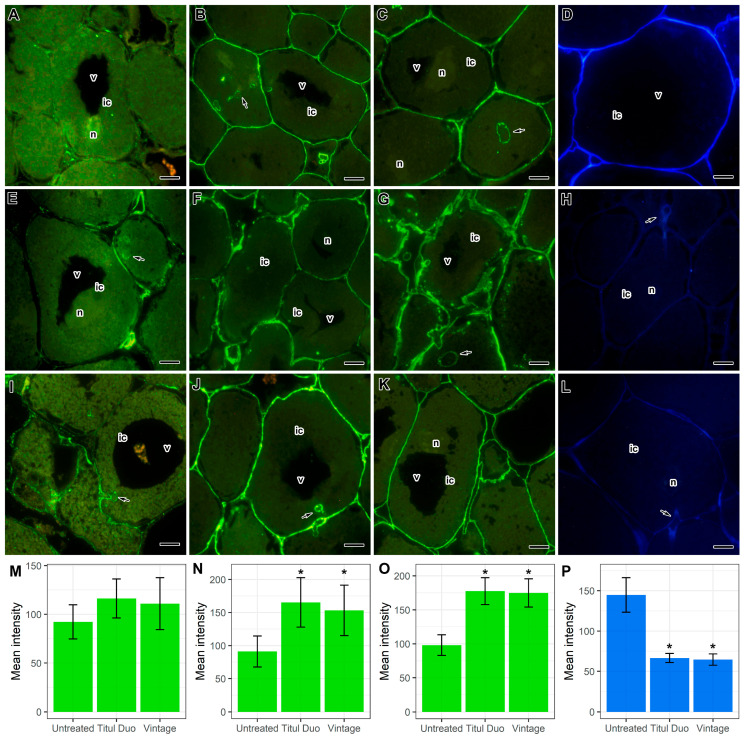
Effect of the fungicide treatment of pea (*Pisum sativum* L.) cv. ‘Frisson’ plants on the cell wall composition in nodule cells. (**A**–**D**) Section of nodules of untreated plants. Treatment with Titul Duo (**E**–**H**) and Vintage (**I**–**L**) with double-concentrated solutions at 10 DAI. (**M**–**P**) Mean fluorescence intensity. (**A**,**E**,**I**,**M**) Immunolocalization of homogalacturonan bound by Ca^2+^ labeled with 2F4 MAb, (**B**,**F**,**J**,**N**) highly methylesterified homogalacturonan labeled with LM20 MAb, (**C**,**G**,**K**,**O**) fucosylated xyloglucan labeled with CCRC-M1 MAb. (**D**,**H**,**L**,**P**) Histochemical staining of cellulose with SCRI Renaissance Stain 2200. The secondary antibodies used were goat anti-mouse (**A**,**C**,**E**,**G**,**I**,**K**) and anti-rat (**B**,**F**,**J**) IgG MAb conjugated with Alexa Fluor 488. ic, infected cell; n, nucleus; v, vacuole. Arrows indicate infection threads. Asterisks indicate statistically significant differences from untreated plants (Tukey’s HSD test, *p*-value < 0.05; *n* = 15), vertical bars represent standard deviation. Bars = 10 µm.

For a more detailed study of the composition of cell walls, the immunocytochemical and histochemical analyses were performed using monoclonal antibodies (MAbs) to various components of cell walls: pectins (homogalacturonans: 2F4 (Figure 10A,E,I) and LM20 (Figure 10B,F,J)), hemicelluloses (fucosylated xyloglucan: CCRC-M1 (Figure 10C,G,K)), and a fluorescent dye to cellulose SCRI Renaissance Stain 2200 (Figure 10D,H,L).

Histochemical analyses showed that in nodules of treated plants, the intensity of fluorescence associated with cellulose microfibrils decreased (Figure 10D,H,L,P), but at the same time, the intensity of epitope labels for highly methylesterified homogalacturonan (Figure 10B,F,J,N), and fucosylated xyloglucan (Figure 10C,G,K,O) was increased. Moreover, especially significant changes were observed when pea plants were treated with the fungicide Titul Duo (Figure 10F,G). However, the intensity of the epitope label for homogalacturonan cross-linked with Ca^2+^ was not significantly increased (Figure 10A,E,I,M).

Thus, the fungicides Titul Duo and Vintage influence the composition of cell walls. It is possible that the increase in the intensity of the label of matrix polysaccharides (pectins and hemicelluloses) in the cell walls of treated plants is associated with their unmasking as a result of a decrease in the number of cellulose microfibrils.

### 2.5. Transcriptome Analysis

Transcriptomic analysis was performed to unravel changes in gene expression associated with a fungicide treatment. Since the most distinct changes were observed in the cv. ‘Frisson’ plants at 20 DAI, and the development of the symbiotic nodule was not so critically disturbed when treated with the fungicide Titul Duo, nodules from such plants were selected for transcriptomic analysis. A total of 55 genes were identified as differentially expressed: 34 upregulated and 21 downregulated (Supplementary Table S1). Gene Ontology enrichment analysis was carried out for these genes, which showed as significantly enriched such “Biological process” terms as “sulfate assimilation”, “polysaccharide catabolic process” and “reproduction” for upregulated genes, while “response to stress” and “polysaccharide biosynthetic process” were significantly enriched for downregulated genes.

## 3. Discussion

Treatment of plants with fungicides increases crop yield due to better plant survival [49,50,51]. Titul Duo and Vintage are systemic combined fungicides, the active ingredients of which belong to the triazole family, that are known to inhibit the synthesis of sterols [12]. Triazoles are broad-spectrum systemic fungicides. They are effective against various species of *Fusarium* [52,53,54], *Rhizoctonia*, *Alternaria*, *Pyricularia*, *Gibberella*, *Botrytis* [55], *Cercospora* [56], *Podosphaera*, *Erysiphe* [57], and *Colletotrichum* [58], inhibiting mycelial growth. Moreover, the role of triazoles in adaptive agriculture is unique since they not only have a pronounced fungicidal effect but also show growth-stimulating [14,59,60] and protective properties against various environmental stresses, such as high temperature [14], drought [23,24,25,26], salinity [22,61], and cooling [62]. Studies of the action of triazoles under various stresses revealed that plant resistance was enhanced due to an increase in the content of chlorophyll and the photosynthetic ability [63], regulation of the activity of enzymes involved in the metabolism of carbon and nitrogen, and changes in the level of endogenous hormones [25,64,65].

It is believed that triazoles are generally not phytotoxic. The most pronounced effect of triazoles on plants is a decrease in height, while the treated plants become greener and more compact [66,67]. Treatment of soybean (*Glycine max* (L.) Merill) plants with uniconazole promoted the accumulation and availability of sucrose and starch content in pods and seeds, thereby increasing the rate of pod setting and soybean yield [60]. However, the application of three different commercial fungicides based on triazole, strobilurin, or carboxamide during the pre-flowering and flowering stage on healthy soybean plants did not affect the physiological traits, pollen grain germination, and yield [17]. In this study, a significant increase in the dry weight of pea shoots under the action of triazole fungicides was not observed (Supplementary Figure S1).

In addition, triazoles have been shown to affect root growth, although this effect can be either inhibitory or stimulatory, depending on the plant and the concentration of the triazole compound used [14]. Paclobutrazol treatment of pea primary roots inhibited root expansion but promoted radial cell expansion [68]. In this study, the dry weight of the roots of fungicide-treated plants decreased compared to the untreated plants when plants were treated at 10 DAI with the highest concentrations of both Titul Duo and Vintage (Supplementary Figure S2A,B). A decrease in the total root weight, a decrease in the number of lateral roots, and root deformation, accompanied by the death of rhizodermal cells and the outer layers of the primary cortex, were also observed under the action of tebuconazole in wheat (*Triticum aestivum* L.) plants [69], hexaconazole [31,33], kitazin [29], naproxen [70], and fluoranthene in pea plants [71].

Despite the positive effects of triazole fungicides on plants, the negative effect of these compounds on various plants, in particular legumes, was also shown [31]. It should be noted that legume-*Rhizobium* symbiosis is very sensitive to stress factors [72]. Treatment of legumes with different fungicides can lead to growth inhibition and chlorosis [48,73,74,75,76]. Plant treatment with certain fungicides reduced or stopped the formation of nodules and disrupted nitrogen fixation. For example, hexaconazole is used to control phytopathogenic fungi [77] but negatively affects biological nitrogen fixation, ureide levels, nitrogen transformation, and the overall yield of legume crops [78,79]. Previously, it was shown that the use of the tetramethylthiuram disulfide (TMTD) fungicide reduced the nodule number in pea laboratory lines Sprint-2 and SGE, as well as in the cv. ‘Finale’ [48]. A decrease in the number of nodules was also reported in pea, lentil, chickpea, and mungbean treated with hexaconazole, with a maximum decrease of 67% compared to untreated plants in pea [33,41]. In the present study, the application of fungicides Titul Duo and Vintage reduced the number of nodules at the highest concentration of fungicides (Figure 3). Moreover, the plants of the pea cv. ‘Frisson’ treated at 10 DAI with double- and tenfold-concentrated solutions of the fungicide Vintage did not form nodules (Figure 2).

Previously, the existence of genotypic variability in the resistance of pea genotypes to the fungicide TMTD [48], alfalfa (*Medicago sativa* L.) cultivars to the fungicide pentachloronitrobenzene [80], and peanut (*Arachis hypogaea* L.) cultivars to various fungicides [81] was shown. On the other hand, the effects of fungicides on chickpea plants did not reveal genotypic differences [82]. In this study, the significant genotypic differences in the nodule ultrastructure between the pea cultivars ‘Finale’ and ‘Frisson’ when treated with fungicides Titul Duo and Vintage were not revealed.

Various changes in physiological parameters have been described when plants are treated with triazole fungicides. For example, hexaconazole reduced the formation of chlorophyll and carotenoids in legumes, such as common bean (*Phaseolus vulgaris* L.) [83]. These fungicides act as polyfunctional inhibitors and have various types of toxic effects on cells, such as the chelation of calcium ions and the formation of mixed disulfide bonds, thereby disrupting membrane transport [33]. The toxicity of fungicides to plants also causes oxidative stress and leads to the accumulation of ROS. However, treatment with paclobutrazol in peanut plants [67,84], uniconazole in soybean plants [65], hexaconazole in mung bean plants [31], and difenoconazole in wheat plants [85] induced an increase in the content of ascorbic acid, alpha-tocopherol, proline, and glutathione, an enhancement in activity of superoxide dismutase, ascorbate peroxidase, and catalase. These antioxidant activities were not enough to remove excess ROS, which then caused oxidative stress and subsequent growth inhibition.

Studies of the action of fungicides on the morphology of legume nodules are extremely scarce. Serious changes in the histological and ultrastructural organization of pea nodules were previously described under the action of high doses of the TMTD fungicide [48]. In the present study, the morphological changes in pea nodules under treatment with low concentrations of widely used foliar fungicides were studied. However, even the recommended concentrations of fungicides caused changes in the structure of pea symbiotic nodules. The histological organization was characterized by changes in the shape of cells in the meristem and infection zone (Figure 5D–I, Supplementary Materials Figure S3D–I, Supplementary Materials Figure S4D–I), as well as the appearance of a senescence zone when treated with fungicides at 10 DAI (Figure 5C, Supplementary Materials Figure S3A–C). Previously, it was shown that infected cells in mung bean nodules treated with hexaconazole were smaller and deformed [31]. The early appearance of the senescence zone was found in common bean and pea nodules under dark shock conditions, after treatment with exogenous nitrates [86], in pea nodules after treatment with cadmium [87,88] and fungicide TMTD [48], as well as in nodules of barrel medic (*Medicago truncatula* Gaertn.) during drought [89].

At the ultrastructural level, the most significant changes were cell wall modifications in response to the treatment of plants with fungicides Titul Duo and Vintage (Figure 6B–D), namely swelling, clearing, and curvature in the meristem and infection zone (Figure 6C), thinning and curvature cell walls of infected cells in the nitrogen fixation zone (Figure 6B,D). With an increase in the concentration of fungicides, negative manifestations were aggravated. It is well known that the cell wall is a cell compartment that performs numerous functions and directly responds to various morphogenic and stress factors [90]. Various cell wall changes have been described in white lupine (*Lupinus albus* L.) nodules after exposure to copper [91] and glyphosate [92], salt [93], and in pea nodules after the fungicide TMTD treatment [48]. The immunocytochemical and histochemical analyses showed that in pea nodules treated with fungicides, the intensity of fluorescence associated with cellulose microfibrils was decreased (Figure 10D,H,L,P), but at the same time, the intensity of the labels to highly methyl esterified homogalacturonan (Figure 10B,F,J,N) and to fucosylated xyloglucan (Figure 10C,G,K,O) was increased. At the same time, especially significant changes were observed when pea plants were treated with the fungicide Titul Duo (Figure 10F,G). The accumulation of pectins and hemicellulose in nodules of yellow lupine (*Lupinus luteus* L.) exposed to drought [94], as well as in the nodules of birdsfoot trefoil (*Lotus corniculatus* L.) impacted with nickel, cobalt, and chromium [95] was previously shown. Application of the herbicide isoxaben reduced the amount of cellulose in callus cultures of common bean [96] and in suspension cell cultures of thale cress (*Arabidopsis thaliana* (L.) Heynh.) [97]. The performed transcriptomic analysis showed that the treatment of pea plants with the fungicide Titul Duo resulted in the downregulation of several genes involved in the cell wall modification (Supplementary Table S1). There were notable changes in the expression of genes for dirigent-like proteins (Psat5g216680, Psat5g216760, Psat7g248760), which are known players in lignin biosynthesis in plants [98]. Moreover, the Psat1g162120 gene, characterized as a coding plant invertase/pectin methylesterase inhibitor, was down-regulated. This family includes proteins that are able to inhibit the activity of two classes of plant carbohydrate enzymes: invertases (which are essential for cellulose biosynthesis, sugar metabolism, and osmotic stress adaptations) and pectin methylesterases (which are involved in the modulation of cell wall stiffness) [99]. It was shown that a high level of de-esterified homogalacturonans was associated with various stresses in nodules: boron deficiency [100], aluminum treatment [101], and inefficient interaction with rhizobia in symbiotic mutants of pea [102,103]. Interestingly, an upregulation of the Psat3g077960 gene with a methyltransferase domain signature was also observed, which also suggests a possible link between the observed alteration in homogalacturonan distribution in treated nodules (Figure 10B,F,J,N) and these expression changes. Downregulation of the Psat1g004960 gene encoding a cellulose synthase-like protein was consistent with the detected decrease in cellulose microfibrils-associated fluorescence (Figure 10D,H,L,P) and the overall thinness of cell walls in treated nodules (Figure 6B–D).

The fungicide treatment affected not only the cell walls but also the infection thread walls. They thickened and swelled (Figure 7B,C), and outgrowths of the wall appeared into the cytoplasm (Figure 7D,E). A fibrillar matrix was distinguished in the infection thread walls (Figure 7F). These abnormalities were more pronounced with an increase in the concentration of fungicides but did not depend on the duration of treatment. At the highest concentration of fungicides, the bacteria trapped in the matrix within the infection thread were degraded (Figure 7C,E). Similar modifications of the infection thread walls and matrix have been described after treatment with the TMTD fungicide [48]. Treatment of *M. truncatula* [104] and pea [105] plants with high concentrations of aluminum and common kidneyvetch (*Anthyllis vulneraria* L.) with zinc and lead [106] led to modifications in the infection thread walls in the form of thickening, swelling, and the appearance of a fibrillar matrix. In addition, the cadmium treatment of pea nodules caused the formation of lateral outgrowths of the infection thread [87]. Interestingly, in the nodules of pea mutant *sym33–2*, bacteria inside infection threads also underwent degradation [107].

In addition to cell walls and infection thread walls, the treatment of pea plants with triazole fungicides exhibited disturbances in the bacteroids and symbiosomes. In infected cells, accumulation of PHB (Figure 8B–D,F), expansion of the peribacteroid membrane (Figure 8B–F), fusion of symbiosomes into multibacteroid symbiosomes (Figure 8D), and transformation of symbiosomes into lytic compartments (Figure 8D–F) were found. When plants were treated with fungicides at the highest concentration, degenerating infected cells with “ghosts” of bacteroids appeared in the nitrogen fixation zone of the nodule (Figure 8F). The same “ghost” bacteroids were observed when lupine plants were treated with glyphosate [92] and mercury [108], *A. vulneraria* with zinc [106], and pea with cadmium [87,88] and TMTD [48]. The accumulation of PHB in the cell is a response to various stresses such as heat shock, ultraviolet radiation, oxidizing agents, and osmotic shock [109]. Previously, we showed the accumulation of PHB in bacteroids in pea plants in response to cadmium [87,88] and the fungicide TMTD [48]. Expansion of the peribacteroid membrane and fusion of symbiosomes in the nodule is a widespread response to various stresses [48,87,88,92,104,105,106,108].

Treatment of pea plants with fungicides Titul Duo and Vintage not only affected the cell walls and infection structures in nodule cells but also led to the appearance of various inclusions in the vacuoles (Figure 5F and Figure 9B–D). Previously, it was shown that electron-dense inclusions, probably of phenolic compounds, appeared in the vacuoles of meristematic cells of pea nodules when plants were treated with high doses of TMTD [48]. Similar inclusions have been described in nodules of *L. corniculatus* under metal stress [95], in pea under saline conditions [110], and in *A. vulneraria* after treatment with zinc and lead [104]. Phenolic compounds were also found in nodules of Chinese liquorice (*Glycyrrhiza uralensis* Fisch. ex DC.) when inoculated with *Mesorhizobium* sp. RCAM3115 [111], as well as big trefoil (*Lotus pedunculatus* Cav.) when inoculated with a rapidly growing strain of rhizobia NZP2037 [112]. The appearance of multivesicular bodies in the vacuoles of nodule cells indicates an increase in the autophagy process in response to stress conditions [113,114]. In the present work, when pea plants were treated with triazole fungicides Titul Duo and Vintage, multivesicular bodies of various shapes were found in the vacuoles of nodule cells (Figure 9D). Such inclusions were described when pea plants were treated with the fungicide TMTD [48] and during the formation of soybean nodules by the citrate synthase mutant strain *Sinorhizobium fredii* USDA257 [115].

## 4. Materials and Methods

### 4.1. Plant Material and Bacterial Strain

The pea (*Pisum sativum* L.) commercial cultivars ‘Finale’ [116] and ‘Frisson’ [117] were used. Both cultivars have a determinate flowering habit composed of white flowers, and they are cultivated in many European countries. ‘Finale’ is a late-ripening cultivar, and ‘Frisson’ is a mid-ripening one. The streptomycin-resistant *Rhizobium leguminosarum* bv. *viciae* strain 3841 was used for inoculation [118]. Bacteria were grown on a solid TY medium [119] at 28 °C with the addition of streptomycin at a concentration of 600–800 µg/L. 

### 4.2. Inoculation and Plant Growth Conditions

Pea seeds of each cultivar were sterilized with concentrated sulfuric acid for 30 min and washed with sterile water 10 times. The seeds were planted in pots with vermiculite immediately after sterilization, and then each seed was inoculated with 1 mL of an aqueous suspension of bacteria (10^7^–10^8^ cells). Plants were grown in vermiculite moistened with a nitrogen-free nutrient solution [120] in a growth chamber (MLR-352H, Sanyo Electric Co., Ltd., Moriguchi, Japan) under controlled conditions: day/night, 16/8; temperature 21 °C; humidity 75%; illumination 280 mol photons m^–2^ s^–1^). An active solution of the fungicide Titul Duo contains 200 g/L of propiconazole and 200 g/L of tebuconazole; Vintage contains 65 g/L of difenoconazole and 25 g/L of flutriafol [9]. Fungicide treatment was carried out with a manual sprayer at 10 and 20 DAI with solutions diluted as follows: Titul Duo—1:500 (recommended by the manufacturer), 1:250 (double-concentrated solution), 1:50 (tenfold-concentrated solution); Vintage—1:200 (recommended by the manufacturer), 1:100 (double-concentrated solution), 1:20 (tenfold-concentrated solution). Plants were harvested 10 days after treatment with fungicides (20- and 30-day-old plants, respectively).

### 4.3. Phenotypic Analysis of Plants and Nodules

Growth and nodule formation parameters were analyzed only for the cv. ‘Frisson’; 20 plants were analyzed. Nodules were counted immediately after washing the plants from vermiculite. For weight measurements, cotyledons were removed, shoots and roots were separated and then dried in a Memmert UF160 oven (Memmert GmbH, Schwabach, Germany) at 40 °C. Pea nodules were photographed using a SteREO Lumar.V12 stereo microscope equipped with an AxioCam MRc 5 camera (Carl Zeiss, Oberkochen, Germany).

### 4.4. Statistical Analysis

Statistical data analysis was carried out using the software STATISTICA version 10 (StatSoft, Tulsa, OK, USA). For phenotypic analysis of plants and nodules, statistically significant differences were assessed using one-way ANOVA (*p* < 0.05) and the least significant difference test (*p* < 0.05). For mean fluorescence intensity, statistically significant differences were assessed using one-way ANOVA (*p* < 0.05) and Tukey’s HSD test (*p* < 0.05).

### 4.5. Electron and Light Microscopy

Nodules (10–15 nodules from 10 plants for each variant) after harvesting were transferred directly into a 2.5% aqueous solution of glutaraldehyde (Sigma-Aldrich, St. Louis, MO, USA) in 0.01 M phosphate buffer (2.48 g/L NaH_2_PO_4_, 21.36 g/L Na_2_HPO_4_, and 87.66 g/L NaCl, pH 7.2). A lateral cut was made on each nodule for better penetration of the fixative. The samples were placed under a vacuum to remove air from the intercellular space and left in a fixative overnight at 4 °C.

Then, the nodules were washed in the buffer four times for 15 min each and postfixed in 1% aqueous solution of osmium tetroxide in 0.1 M phosphate buffer for 1 h. The nodules were then dehydrated in a series of increasing concentrations of ethanol followed by two changes to 100% acetone, as described previously [121]. Dehydrated samples were gradually infiltrated with epoxy resin Eponate 12 (Ted Pella, Inc., Redding, CA, USA). All these procedures were performed in the EM TP Tissue Processor (Leica Microsystems, Vienna, Austria) at 21 °C. The samples were transferred for embedding to small plastic containers with fresh resin, which were polymerized at 60 °C for 48 h.

For light microscopy, semi-thin sections (1 µm) obtained on a Leica EM UC7 ultra-microtome (Leica Microsystems) were placed on slides and stained with methylene blue–azure II [122] at 60 °C for 20 min. Sections were then placed in a drop of xylene and embedded in the EUKITT^®^ Mounting Medium (Electron Microscopy Sciences, Hatfield, PA, USA). Sections were analyzed using an Axio Imager.Z1 microscope (Carl Zeiss). Photographs were taken with an Axiocam 506 digital camera (Carl Zeiss).

For transmission electron microscopy, ultrathin sections (90–100 nm thick) were cut with a Leica EM UC7 ultramicrotome (Leica Microsystems) using a diamond knife (Diatome, Nidau, Switzerland). The sections were collected on copper grids coated with 4% formvar and carbon. Sections were counterstained with 2% aqueous uranyl acetate for 30 min followed by lead citrate for 1 min in the automatic contrasting system for ultrathin sections EM AC20 (Leica Microsystems) at 21 °C. All solutions were filtered before use, and filter-sterilized deionized water was used throughout the experiment. Nodule tissues were examined using a JEM-1400 EM transmission electron microscope (JEOL Ltd., Tokyo, Japan) at 80 kV. Electron micrographs were taken with a Veleta CCD camera (Olympus, Münster, Germany). 

### 4.6. Fluorescence Microscopy

For immunofluorescence microscopy, semi-thin sections (1 µm) obtained on a Leica EM UC7 ultratome were incubated in ABB blocking buffer (5% BSA, 0.1% cold water fish skin gelatin (CWFS), 5–10% normal goat serum, 15 mM NaN_3_ in PBS, pH 7.4) for 1 h at room temperature. Then nodule sections were incubated with primary antibodies diluted 1:20 in 3% BSA in PBS at 37 °C for 1 h. The samples were washed again in 3% BSA in PBS (pH 7.2) two times for 20 min each. The incubation with the secondary antibodies to the corresponding gamma globulin conjugated to AlexaFluor 488 (Molecular Probes, Thermo Fisher Scientific, Waltham, MA, USA) in 3% BSA in PBS (diluted 1:100) was conducted for 1 h at 37 °C. Then, samples were washed in a 3% BSA solution in PBS two times for 20 min. After staining complete drying, sections were covered with a drop of ProLong Gold Antifade reagent (Molecular Probes, Thermo Fisher Scientific).

The fluorescent dye SCRI Renaissance Stain 2200 (Renaissance Chemicals, North Duffield, UK) was used to detect cellulose [123]. Semi-thin sections (1 µm) were stained with a dye (diluted 1:1000) for 20 min, washed with distilled water, and mounted in the ProLong Gold^®^ antifade reagent (Molecular Probes, Thermo Fisher Scientific). Sections were examined under a fluorescence microscope using a DAPI filter.

The following MAbs were used as primary antibodies: LM20 for high methylester homogalacturonan [124], 2F4 for a dimeric association of homogalacturonan chains through Ca^2+^ [125], CCRC-M1 fucosylated xyloglucan [126]. Sections were analyzed using an Axio Imager.Z1 microscope (Carl Zeiss). Photos were taken using a digital camera Axiocam 506 (Carl Zeiss).

Image analysis was performed using the program ImageJ [127] to detect fluorescence intensities. During the mathematical processing of fluorescent images, the areas of the presence of a signal and its absence were selected, the average fluorescence intensity was identified, and the average fluorescence intensity of the signal was normalized to the average intensity of the area without a signal.

### 4.7. Transcriptomic Analysis

For the RNA-seq analysis, nodules were harvested on ice and frozen in liquid nitrogen. RNA extraction was performed using the RNAeasy Plant Mini Kit (Qiagen, Hilden, Germany). The concentration of the extracted RNA was measured using Qubit 2.0 (Invitrogen, Waltham MA, USA). 

The extracted RNA was used for the preparation of libraries using the RapidMACE kit (GenXPro GmbH, Frankfurt, Germany) according to the manufacturer’s recommendations. Libraries were sequenced with Illumina HiSeq 2500 by the Macrogen company (Seoul, Republic of Korea). Quality assessment of the obtained reads, filtering, mapping to the reference genome, and subsequent bioinformatics analysis was performed as previously described [128].

## 5. Conclusions

Thus, symbiotic pea nodules are highly sensitive to the phytotoxic action of triazole fungicides Titul Duo and Vintage. All studied concentrations of fungicides, including those recommended by the manufacturer, caused structural changes in cell walls, infection structures (infection threads and symbiosomes), as well as increased autophagy processes. The data obtained by transcriptomic analysis indicate the modification of cell walls and coincide well with the data of light and electron microscopic studies. In the present study, no differences in the nodule ultrastructure between pea cultivars when treated with fungicides were revealed. Agricultural companies strive to reduce the cost of pesticides; therefore, more preparations of complex and combined action will appear in the market. A deeper understanding of the negative effects of agricultural pesticides is needed to create new and optimized strategies for growing legumes.

## Data Availability

The data presented in this study are openly available at NCBI SRA under the accession number PRJNA953817.

## Supplementary Materials

The following supporting information can be downloaded at: https://www.mdpi.com/article/10.3390/ijms24108646/s1.
