# Supplementary material for: Effect of Triazole Fungicides Titul Duo and Vintage on the Development of Pea (Pisum sativum L.) Symbiotic Nodules
Source: Int J Mol Sci. 2023 May 12;24(10):8646. doi: 10.3390/ijms24108646 (PMC10217885; doi:10.3390/ijms24108646)
Supplement: Supplementary file 1 [file ijms-24-08646-s001.zip › Supplementary_Gorshkov.pdf]

Electronic Supplementary Material

Effect of triazole fungicides Titul Duo and Vintage on the development of symbiotic pea nodules (*Pisum sativum* L.)

Artemii P. Gorshkov[1], Pyotr G. Kusakin[1], Yaroslav G. Borisov[2], Anna V. Tsyganova[1] and Viktor E. Tsyganov[1, 3,*]

[1] All-Russia Research Institute for Agricultural Microbiology, Laboratory of Molecular and Cell Biology, Saint Petersburg, Russia; artemius1993@yandex.ru (A.P.G.); kussakin@gmail.com (P.G.K.); avtsyganova@arriam.ru (A.V.T.)

[2] Research Resource Centre "Molecular and Cell Technologies", Saint Petersburg State University, 199034 Saint Petersburg, Russia; gis.ib88@gmail.com (Y.G.B.)

[3] Saint Petersburg Scientific Center RAS, Universitetskaya Embankment 5, Saint Petersburg 199034, Russia

*Author for correspondence:

Viktor E. Tsyganov

Tel: +7 812 4705100

E-mail: vetsyganov@arriam.ru

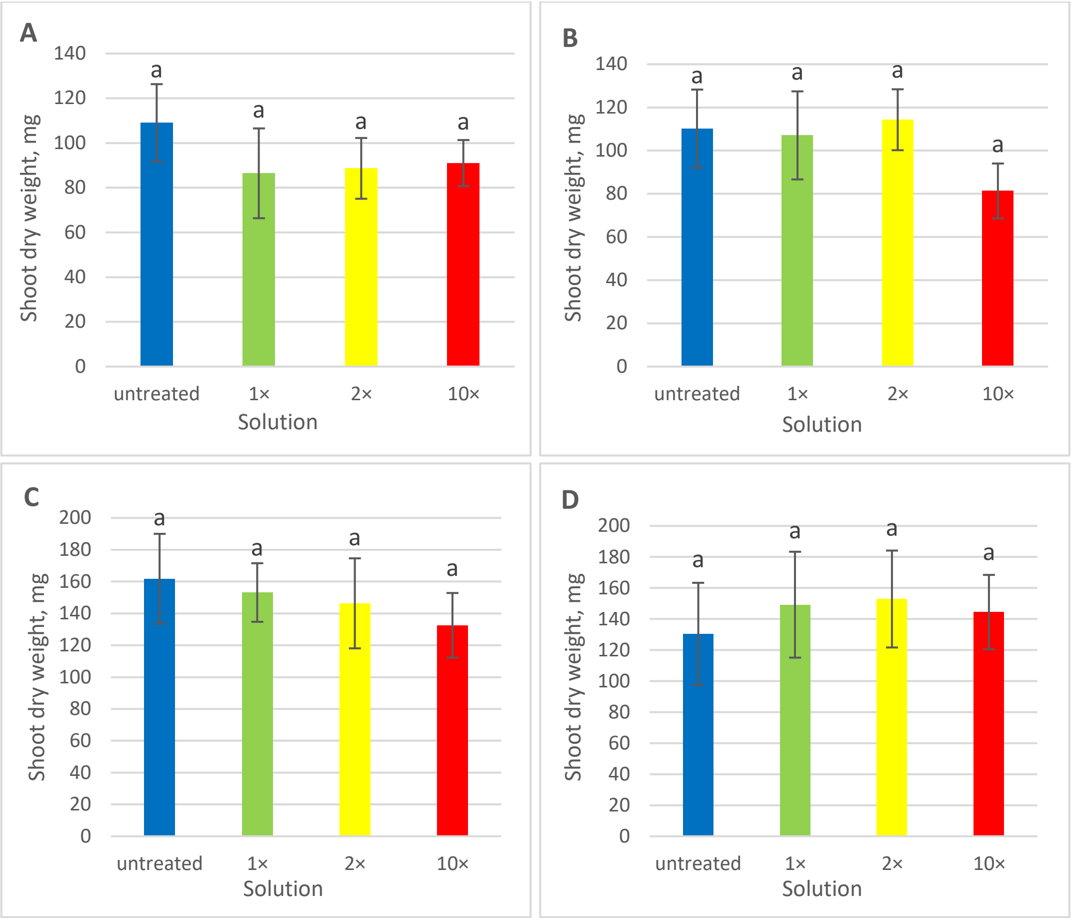

Figure S1. Mean dry weight of shoots of pea (*Pisum sativum* L.) cv. 'Frisson' treated with recommended by the manufacturer (**1×**), double- (**2×**), and tenfold-concentrated (**10×**) solutions of fungicides Titul Duo (**A,C**) and Vintage (**B,D**). (**A,B**) Fungicide treatment at 10 DAI. (**C,D**) Fungicide treatment at 20 DAI. Different letters indicate at groups with significant difference according to the least significant difference test ($p < 0.05$; $n = 20$). Vertical bars represent standard deviation.

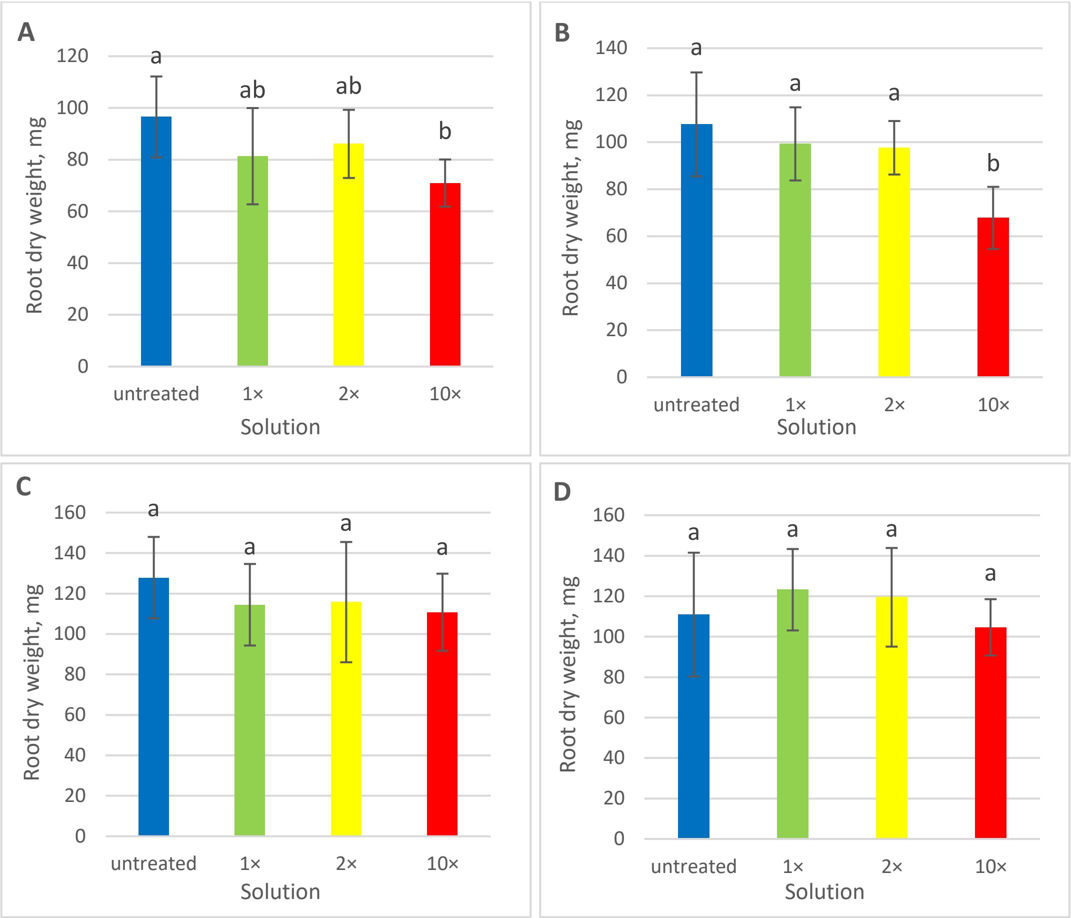

Figure S2. Mean dry weight of roots of pea (*Pisum sativum* L.) cv. 'Frisson' treated with recommended by the manufacturer (**1×**), double- (**2×**), and tenfold-concentrated (**10×**) solutions of fungicides Titul Duo (**A,C**) and Vintage (**B,D**). (**A,B**) Fungicide treatment at 10 DAI. (**C,D**) Fungicide treatment at 20 DAI. Different letters indicate at groups with significant difference according to the least significant difference test ($p < 0.05$; $n = 20$). Vertical bars represent standard deviation.

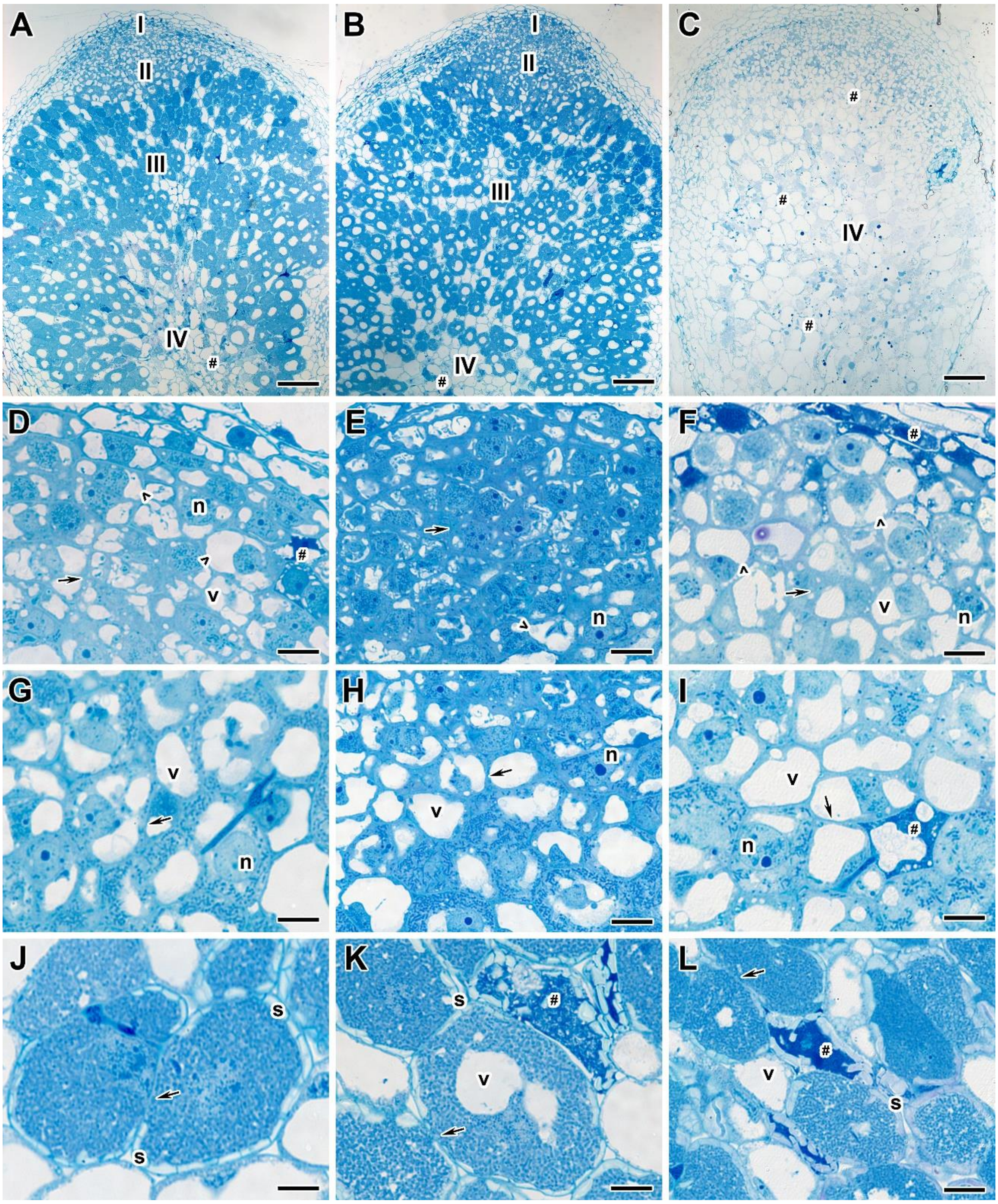

Figure S3. Histological organization of the nodules of pea (*Pisum sativum* L.) cv. 'Frisson' treated with fungicide Vintage at 10 DAI. (**A**,**D**,**G**,**J**) Treatment with fungicide at the concentration recommended by the manufacturer. (**B**,**E**,**H**,**K**) Treatment with a double-concentrated solution of fungicide. (**C**,**F**,**I**,**L**) Treatment with a tenfold-concentrated solution of fungicide. (**A**–**C**) Longitudinal section of a nodule. (**D**–**F**) Nodule meristematic cells. (**G**–**I)** Cells in the infection zone. (**J**–**L**) Infected cells in the nitrogen fixation zone. I, meristem; II, infection zone; III, nitrogen fixation zone; IV, senescence zone; n, nucleus; v, vacuole; #, degrading cells; s, starch accumulation. Arrows indicate a barely visible cell wall between infected cells, empty arrowheads indicate vacuole fusion. Bars (**A**–**C**) = 100 μm, (**D**–**M**) = 10 μm.

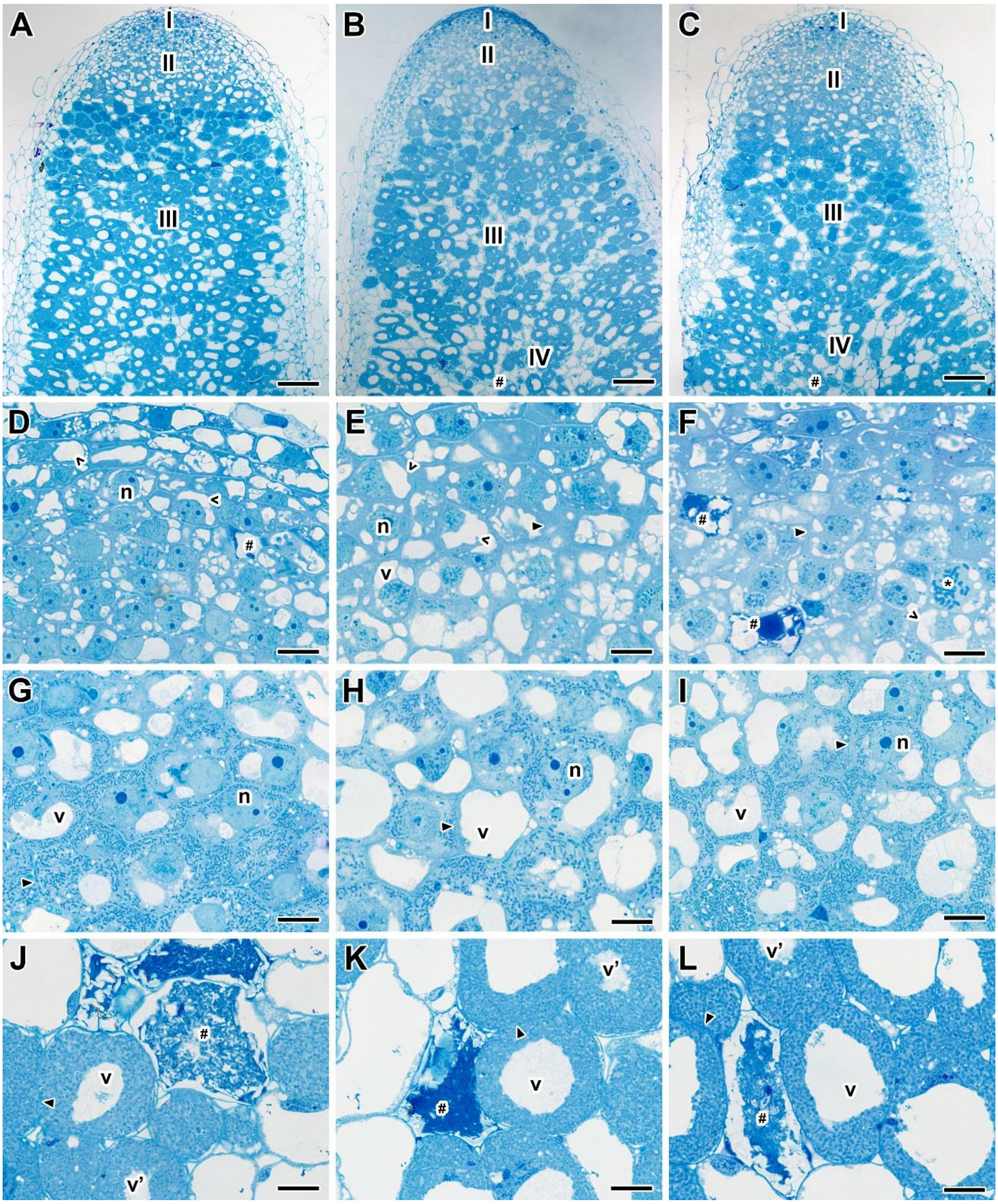

Figure S4. Histological organization of the nodules of pea (*Pisum sativum* L.) cv. 'Frisson' plants treated at 20 DAI with recommended by the manufacturer (**A,D,G,J**), double- (**B,E,H,K**), and tenfold-concentrated (**C,F,I,L**) solutions of Titul Duo (**C,F,G,I,J,K**) and Vintage (**A,B,D,E,H,L**). (**A–C**) Longitudinal section of a nodule. (**D–F**) Nodule meristematic cells. (**G–I)** Cells in the infection zone. (**J–L**) Infected cells in the nitrogen fixation zone. I, meristem; II, infection zone; III, nitrogen fixation zone; IV, senescence zone; n,nucleus; v, vacuole; v′, destroyed tonoplast; *, metaphase plate; #, degrading cells. Arrows indicate a barely visible cell wall between infected cells, empty arrowheads indicate vacuole fusion. Bars (**A–C**) = 100 μm, (**D–M**) = 10 μm.
